# Acupuntura na Hipertensão Arterial e Disfunção Endotelial: Um Ensaio Clínico Randomizado

**DOI:** 10.36660/abc.20240596

**Published:** 2025-02-07

**Authors:** Amanda Rodrigues Bitencourt, Ana Luiza Lima Sousa, Priscila Valverde de Oliveira Vitorino, Mikaelle Costa Correia, Alexandre Massao Yoshizumi, Weimar Kunz Sebba Barroso

**Affiliations:** 1 Universidade Federal de Goiás Unidade de Hipertensão Arterial Goiânia GO Brasil Universidade Federal de Goiás – Unidade de Hipertensão Arterial, Goiânia, GO – Brasil; 2 Pontifícia Universidade Católica de Goiás Escola de Ciências Sociais e da Saúde Goiânia GO Brasil Pontifícia Universidade Católica de Goiás – Escola de Ciências Sociais e da Saúde, Goiânia, GO – Brasil; 3 Hospital Israelita Albert Einstein São Paulo SP Brasil Hospital Israelita Albert Einstein, São Paulo, SP – Brasil

**Keywords:** Acupuntura, Hipertensão, Pré-Hipertensão, Endotélio

## Introdução

A hipertensão arterial (HA) é um dos principais fatores de risco modificáveis para doenças cardiovasculares,^1^ por acarretar alterações como aumento da rigidez arterial e disfunção endotelial já em fases iniciais.^2^

A hiperatividade simpática e a ativação do sistema renina-angiotensina-aldosterona (SRAA) destacam-se como mecanismos fisiopatogênicos da HA, além da produção exacerbada de espécies reativas de oxigênio (ROS) e desequilíbrios na biodisponibilidade de óxido nítrico (NO).^3^ O aumento da permeabilidade endotelial presente em estados de baixa produção de NO explicam a correlação da HA com aterosclerose e tornam a análise da função endotelial uma ferramenta útil na avaliação do seu curso clínico.^1,4,5^

Apesar do vasto arsenal terapêutico disponível para seu tratamento, a HA ainda é mal controlada em todo mundo^1^ e as recomendações de tratamento não farmacológico devem ser feitas para todo paciente hipertenso.^1,3,6^ Algumas publicações apontam para o efeito benéfico da acupuntura (ACP) em pacientes hipertensos.^7–16^ O estímulo álgico gerado pela agulha alcança os neurônios pré-ganglionares simpáticos da medula ventrolateral rostral, bloqueia o reflexo autonômico simpatoexcitatório visceral e reduz o espasmo do músculo liso.^7,17–19^ Além disso, o estímulo inibe a ativação simpática mediada pela ação central do SRAA,^20,21^ reduz a secreção e atividade da renina e de receptores de angiotensina II^19,20^ e dos níveis de ROS, e ativa a NO sintase endotelial, levando ao aumento da biodisponibilidade de NO, à vasodilatação e à melhora da disfunção endotelial,^7,21–24^ contribuindo para o efeito da ACP no controle pressórico.^7,17–24^

Diante disso, este artigo descreve o método de um ensaio clínico randomizado que avaliará os efeitos antes e após 24 sessões da ACP na pressão arterial (PA) e PA central (PAC), na velocidade de onda de pulso (VOP) e na dilatação fluxo-mediada (FMD) em pacientes pré-hipertensos ou hipertensos estágio 1 em baixo risco cardiovascular.

## Métodos

### Tipo e local do estudo

Ensaio clínico randomizado, placebo controlado, simples cego e unicêntrico, cuja coleta de dados será realizada no centro de pesquisa da Unidade de Hipertensão Arterial (UHA) da Universidade Federal de Goiás (UFG).

O estudo está registrado no Registro Brasileiro de Ensaios Clínicos sob o número RBR-9qnxb9z e será realizado segundo recomendações do protocolo STRICTA/CONSORT.^25^ O estudo foi aprovado pelo Comitê de Ética em Pesquisa do Hospital das Clínicas da UFG, sob o número 6.244.770.

### População, amostra e amostragem

A população do estudo será composta por adultos pré-hipertensos ou hipertensos estágio 1 de baixo risco cardiovascular, sem medicações anti-hipertensivas.

Serão excluídos pacientes com HA estágios 2 ou 3, com moderado ou alto risco cardiovascular, diabéticos, nefropatas, com história de doença arterial coronariana e/ou cerebrovascular, insuficiência cardíaca, tireoideopatias, condições que prejudiquem a condução aferente e eferente do estímulo nervoso, doenças psiquiátricas não controladas, discrasias sanguíneas graves, e lesões de pele e/ou condições que impossibilitem acesso aos acupontos. Gestantes e lactantes, pacientes com ou aqueles que tenham recebido tratamento anti-hipertensivo medicamentoso nos últimos seis meses ou ACP em qualquer momento da vida também serão excluídos.

A amostra foi calculada considerando a comparação de médias de dois grupos independentes de pelo menos 3 mmHg para a pressão sistólica, com um alfa de 5%, poder do teste de 90% e perda amostral de 10%; obtendo uma amostra de 25 participantes em cada grupo.^10^

### Recrutamento e randomização dos pacientes

Os participantes serão selecionados de um banco de dados próprio da UHA, composto por encaminhamento e por campanhas de rastreamento, e avaliados em três visitas.

### Visita inicial (VI)

Todos os pacientes serão avaliados por médica cardiologista e acupunturiatra por meio de anamnese e exame físico. A confirmação do estágio da HA será feita por medida da PA conforme orientações da Diretriz Brasileira de Hipertensão Arterial,^1^ utilizando o aparelho oscilométrico automático OMRON 1120.

Após preenchimento de critérios de inclusão e aceite em participar do estudo, todos os pacientes assinarão um Termo de Consentimento Livre e Esclarecido. Na sequência serão randomizados pelo site *www.randomization.com* para um dos dois grupos: intervenção (G1) e controle (G2). Todos os pacientes serão submetidos à avaliação da função endotelial por FMD e à medição da PAC e da VOP.

**Figura 1:**
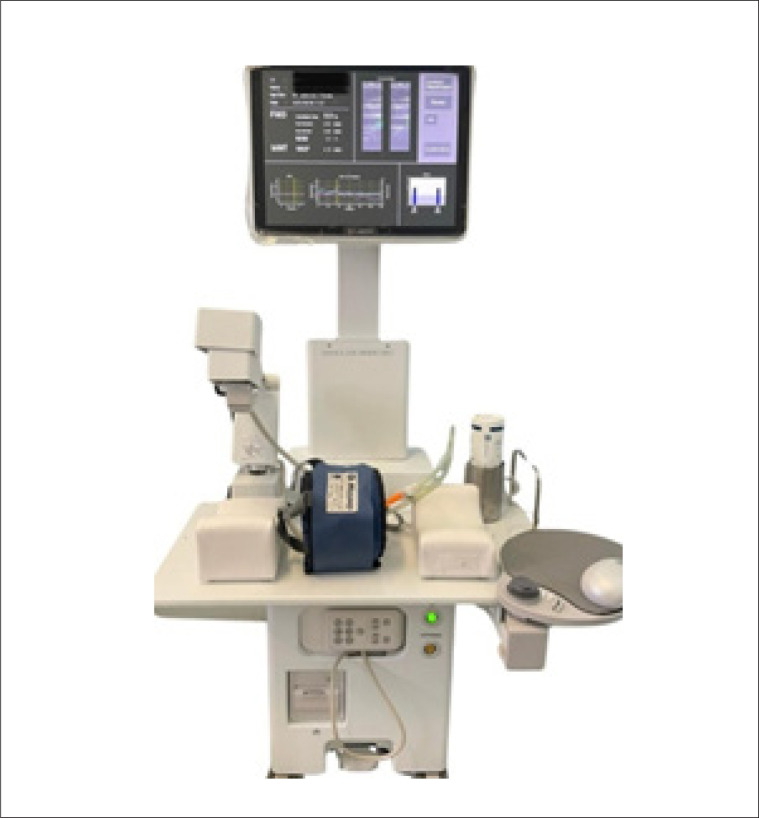
Aparelho de ultrassonografia de alta resolução, UNEX EF38G.

A avaliação da função endotelial pela FMD será realizada com o uso de um aparelho de ultrassonografia de alta resolução UNEX EF38G, o qual possui um dispositivo robótico que faz a varredura da artéria braquial no braço do paciente de forma automática, uniforme e precisa (Figura 1). O exame será realizado conforme orientações de diretrizes.^26–28^

*Fonte: Acervo próprio.*

As medidas da PAC e do grau de rigidez arterial serão realizadas pelo Mobil-OGraph® (IEM, Stolber, Alemanha), método oscilométrico que fornece PAC, VOP e *Augmentation* Index.^29,30^

### Intervenção (G1) e controle (G2)

**Figura 2:**
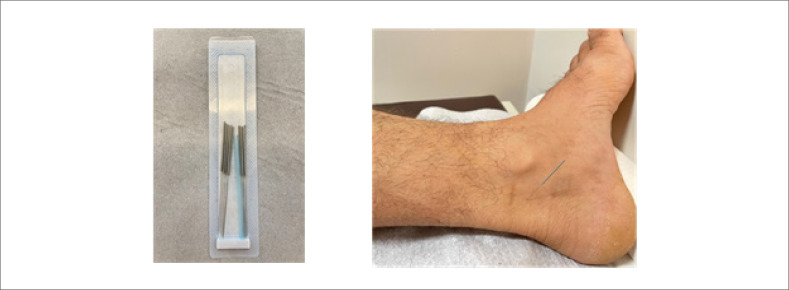
Agulhas de Acupuntura de aço inoxidável.

**Figura 3:**
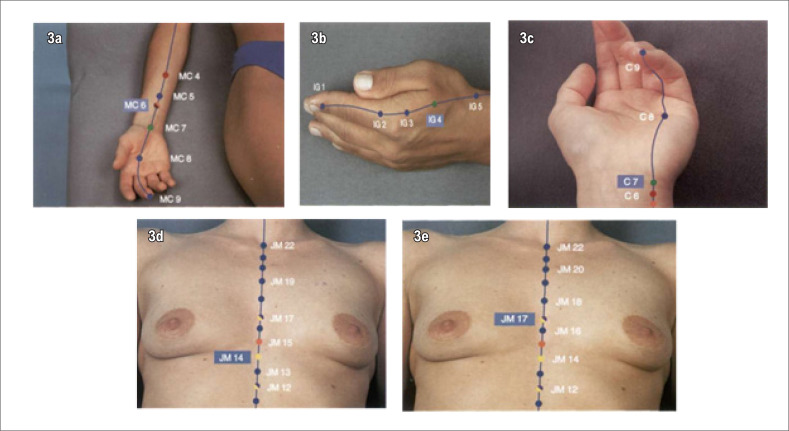
Acupontos: 3a. PC6 (MC6- Neiguan); 3b. IG4 (Hegu); 3c. C7 (Shenmen); 3d. VC14 (JM14 - Juque); 3e. VC17(JM17 - Shanzhong).

Nos pacientes do G1 será realizada a inserção de oito agulhas de ACP de aço inoxidável estéreis, tamanho 0,25x30 mm (Figura 2), com técnica concordante com princípios da Medicina Tradicional Chinesa^31^ e orientações do Colégio Médico Brasileiro de Acupuntura. O procedimento será realizado por médica acupunturiatra, em oito acupontos: PC6, IG4, C7, VC14, VC17, E36, F3, R3 (Figuras 3 e 4).

*Fonte: Acervo próprio.*

*Fonte: Adaptado de Lian et al*.^33^

**Figura 4:**
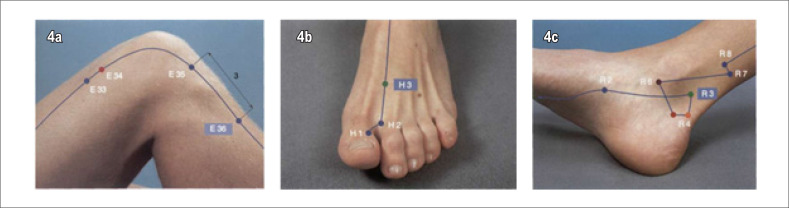
Acupontos: 4a. E36(Zusanli); 4b. F3 (H3 - Taichong); 4c. Acuponto R3 (Taixi).

*Fonte: Adaptado de Lian et al*.^33^

**Figura 5:**
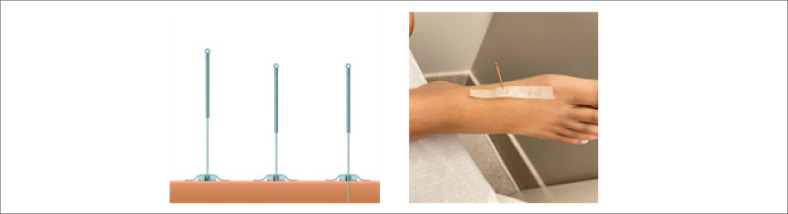
Agulha de Streitberger. Primeira e segunda agulhas não penetrantes.

Pacientes do G2 serão tratados com oito agulhas não penetrantes (agulhas de Streitberger)^32^ distribuídas em tronco e membros, pela mesma médica, em oito não acupontos (Figura 5).

*Fonte:*https://www.acupunctureworld.com/*acervo próprio*.

Todos os participantes receberão duas sessões semanais, de 30 minutos, com intervalo entre elas não superior a três dias, por doze semanas consecutivas, totalizando 24 sessões. Os participantes usarão venda ocular individual para que não identifiquem visualmente em qual grupo estão alocados.

### Visita final e visita de acompanhamento (VP)

Todos os pacientes participarão da visita final (VF) que será realizada após 24 sessões de ACP, e da visita de acompanhamento após 30 dias da VF. Em ambas, serão realizadas avaliação clínica e medidas da PA, da PAC, VOP e avaliação da função endotelial por FMD.

Na visita de VP, todos os pacientes serão questionados quanto ao tipo de agulha que acreditam terem recebido durante as sessões – agulha que penetra a pele (agulha de ACP) ou agulha que não penetra a pele (agulha placebo de Streitberger).

### Análise estatística dos dados

As variáveis categóricas serão apresentadas com seus valores de frequência e proporção. Todas as análises de associação dessas variáveis deverão ser feitas com teste do qui-quadrado ou teste exato de Fisher, conforme adequação do tamanho da amostra. As variáveis quantitativas contínuas serão analisadas primeiramente quanto à normalidade da distribuição com aplicação do teste de Shapiro-Wilk. Serão aplicados testes paramétricos, como o teste t de Student ou o teste de Man-Whitney, e a análise univariada (ANOVA) ou o teste de Kruskal-Wallis, segundo a distribuição encontrada. Todos os testes deverão considerar um erro tipo I de 5% e 80% para erro Tipo II, com intervalo de confiança de 95%.

## Conclusão

Se a eficácia da ACP para redução da PA e melhora da função endotelial for comprovada, a terapia poderá ser recomendada como modalidade terapêutica não-farmacológica para hipertensão em diretrizes futuras.
