# Supplementary material for: Acupuntura na Hipertensão Arterial e Disfunção Endotelial: Um Ensaio Clínico Randomizado
Source: Arq Bras Cardiol. 2025 Feb 7;122(1):e20240596. [Article in Portuguese] doi: 10.36660/abc.20240596 (PMC11884666; doi:10.36660/abc.20240596)
Supplement: Translation

# Translation: Acupuncture for Essential Hypertension and Endothelial Dysfunction: A Randomized Clinical Trial

**DOI:** 10.36660/abc.20240596i

**Authors:** Amanda Rodrigues Bitencourt, Ana Luiza Lima Sousa, Priscila Valverde de Oliveira Vitorino, Mikaelle Costa Correia, Alexandre Massao Yoshizumi, Weimar Kunz Sebba Barroso

**Affiliations:** 1 Goiânia GO Brazil Universidade Federal de Goiás – Unidade de Hipertensão Arterial, Goiânia, GO – Brazil; 2 Goiânia GO Brazil Pontifícia Universidade Católica de Goiás – Escola de Ciências Sociais e da Saúde, Goiânia, GO – Brazil; 3 São Paulo SP Brazil Hospital Israelita Albert Einstein, São Paulo, SP – Brazil

**Keywords:** Acupuncture, Hypertension, Prehypertension, Endothelium

## Introduction

Arterial hypertension (AH) is one of the main modifiable risk factors for cardiovascular diseases,^1^ as it causes increased arterial stiffness and endothelial dysfunction since early stages.^2^

Sympathetic hyperactivity and activation of the renin-angiotensin-aldosterone system (RAAS) are among the major pathophysiological mechanisms of AH, in addition to an overproduction of reactive oxygen species (ROS) and imbalances in the nitric oxide (NO) availability.^3^ The increase in endothelial permeability in response to low NO synthesis explains the correlation of AH with atherosclerosis and makes the analysis of endothelial function a useful tool for clinical assessment.^1,4,5^

Despite the wide availability of therapeutic options, AH is still poorly controlled worldwide^1^ and recommendations for non-pharmacological therapy should be given to hypertensive patients.^1,3,6^ Some evidence has suggested the beneficial effects of acupuncture (ACP) in hypertensive patients.^7–16^ The pain stimulus generated by the needle insertion reaches sympathetic preganglionic neurons of the rostral ventrolateral medulla, blocks autonomic visceral, sympathoexcitatory reflexes and reduces smooth muscle spasm.^7,17–19^ In addition, it inhibits sympathetic activation mediated by the RAAS system,^20,21^ reduces the release and activity of renin and angiotensin II receptors^19,20^ and levels of ROS, and activates endothelial NO synthase, increasing NO bioavailability and vasodilation and improving endothelial dysfunction,^7,21,24^ thereby contributing to the effects of ACP on blood pressure control.^7,17–24^

This article describes the methodology of a randomized clinical trial that will evaluate the effects of ACP on peripheral blood pressure, central blood pressure (CBP), pulse wave velocity (PWV) and flow-mediated dilation (FMD). These parameters were measured before and after 24 sessions of ACP in prehypertensive and stage 1 hypertensive patients at low cardiovascular risk.

## Methods

### Study type and location

This will be a randomized, placebo-controlled, single-blind., single-center clinical trial; data will be collected at the research center of the Arterial Hypertension Unit of Goias Federal University (UFG).

The study is registered at the Brazilian Registry of Clinical Trials (RBR-9qnxb9z) and will be conducted following the STRICTA/CONSORT recommendations.^25^ The study was approved by the ethics committee of the UFG general hospital (approval number 6.244.770).

### Population and sampling

Study population will consist of prehypertensive or stage 1 hypertensive adults at low cardiovascular risk that are not taking antihypertensive medications.

Patients at stage 2 or 3 AH, at moderate/high cardiovascular risk, and patients with diabetes, history of coronary arterial disease, cerebrovascular disease, heart failure thyroid diseases, conditions that may affect afferent and efferent innervations, uncontrolled psychiatric disorders, severe blood dyscrasias, and skin lesions or other conditions that make it impossible to access the ACP points will be excluded. Pregnant and lactating women, and patients who have received antihypertensive treatment in the previous six months or have ever received ACP will also be excluded.

Sample size was calculated considering the comparisons of the means of two independent groups of at less 3 mmHg for systolic pressure, with an alpha of 5%, power of the test of 90% and loss to follow-up of 10%, yielding a sample of 25 participants in each group.^10^

### Patient recruitment and randomization

Patients will be selected from a database of the Arterial Hypertension Unit of UFG, composed by patient referral or screening campaigns, and will be assessed during three visits.

### Visit 1

All patients will be seen by a cardiologist and a physician acupuncturist through history taking and physical examination. The stage of AH will be confirmed by measurement of blood pressure following the Brazilian guidelines on AH,^1^ using an automated oscillometric device (OMRON 1120).

After fulfilling the inclusion criteria and agreeing to participate in the study, all patients will sign an informed consent form. Subsequently, the patients will be randomized using *www.randomization.com* and allocated to either intervention group (G1) or control group (G2). Then, all patients will be assessed for endothelial function by FMD, and for CBP and PWV.

Assessment of endothelial function by FMD will be conducted using a high-resolution ultrasound device (UNEX ED38G), which is equipped with a robotic probe that scans through patient's brachial artery automatically, in a uniform and precise way (Figure 1). The test will be conducted following guidelines’ recommendations.^26–28^

**Figure 1:** High-resolution ultrasound equipment, UNEX EF38G.

Source: Authors’ archive.

Analysis of arterial stiffness and CBP will be performed using the Mobil-OGraph® (IEM, Stolber, Germany), which is an oscillometric method that estimates CBP, PWV and Augmentation Index.^29,30^

### Intervention (G1) and control (G2)

In the G1 patients, eight stainless steel, sterile needles (0.25 x 0.30 mm) (Figure 2) will be inserted in eight points – PC6, IG4, C7, VC14, VC17, E36, F3, R3 (Figures 3 and 4). The technique will be conducted by a physician acupuncturist, following the principles of traditional Chinese medicine^31^ and the Brazilian Acupuncture Medical College's recommendations.

**Figure 2:** Stainless steel needles.

Source: Authors’ archive.

**Figure 3:** Acupuncture points: 3a. PC6 (MC6- Neiguan); 3b. IG4 (Hegu); 3c. C7 (Shenmen); 3d. VC14 (JM14 - Juque); 3e. VC17(JM17 - Shanzhong).

Adapted from the Lian et al.^33^

**Figure 4:** Acupuncture points: 4a. E36 (Zusanli); 4b. F3 (H3 - Taichong); 4c. R3 (Taixi).

Adapted from the Lian et al.^33^

Patients in the G2 group will be treated with eight placebo needles (Streitberger needles) that do not penetrate the skin^32^ in eight non-ACP points throughout the trunk and limbs. The technique will be performed by the same physician (Figure 5).

**Figure 5:** Streitberger needle. First and second are non penetrate neddles.

Source: https://www.acupunctureworld.com/ authors’ archive.

All patients will receive two 30-minute sessions weekly, with an interval of up to three days between them, for 12 consecutive weeks (total of 24 sessions). Each participant will be blindfolded during the procedure as they will be blinded for group allocation.

### Final visit and follow-up visit

All patients will be seen in a final visit (FV) after 24 ACP sessions and in a follow-up visit 30 days after the FV. In both visits, participants will undergo clinical assessment, blood pressure, CBP and PWV measurements, and FMD for assessment of endothelial function.

During the follow-up visit, all patients will be asked about the type of needle they believed that they received during the sessions – ACP needle (that penetrates the skin) *vs*. non-ACP/placebo needle (Streitberger needle, which does not penetrate the skin).

### Statistical analysis

Categorical variables will be expressed as frequencies and proportions. Association analysis of these variables should be done by chi-square test or Fisher exact test, according to sample size. Continuous quantitative variables will be first assessed for normality distribution using the Shapiro-Wilk test. Then, parametric tests like the Student's t-test or the Mann-Whitney test and the ANOVA or the Kruskal-Wallis test will be applied according to data distribution. All tests should consider a type I error of 5% and a type II error of 80%, with a 95% confidence interval.

## Conclusion

If the effectiveness of ACP in reducing blood pressure and improving endothelial function is proven, it can be suggested as a non-pharmacological therapy for hypertension in future guidelines.
